# Green carbon from bagasse for uniform coating of Fe_2_O_3_ nanoparticles toward high-capacity and long-life lithium-ion battery anodes

**DOI:** 10.1039/d5ra07487h

**Published:** 2025-11-13

**Authors:** Quoc Hai Nguyen, Chanwoo Park, Sang The Chung, Thu Huyen Nguyen Thi, To Giang Tran, Jong-Seong Bae, Tuan Loi Nguyen, Jaehyun Hur

**Affiliations:** a Group of Applied Research in Advanced Materials for Sustainable Development, Faculty of Applied Sciences, Ton Duc Thang University Ho Chi Minh City Vietnam nguyenquochai@tdtu.edu.vn; b School of Chemical, Biological, and Battery Engineering, Gachon University Seongnam Gyeonggi 13120 Republic of Korea chanwoo5061@gachon.ac.kr jhhur@gachon.ac.kr; c Faculty of Applied Sciences, Ton Duc Thang University Ho Chi Minh City Vietnam chungthesang.st@tdtu.edu.vn nguyenthithuhuyen.st@tdtu.edu.vn; d Institute of Research and Development, Duy Tan University Da Nang Vietnam trantogiang@duytan.edu.vn; e School of Engineering & Technology, Duy Tan University Da Nang Vietnam; f Yeongnam Regional Center, Korea Basic Science Institute Busan 46742 Korea jsbae@kbsi.re.kr; g Institute of Fundamental and Applied Sciences, Duy Tan University Ho Chi Minh City 70000 Vietnam nguyentuanloi@duytan.edu.vn; h Faculty of Natural Sciences, Duy Tan University Da Nang City 50000 Vietnam

## Abstract

Sustainable high-performance anodes are essential for next-generation Li-ion batteries (LIBs). In this study, we develop an Fe_2_O_3_@C composite in which Fe_2_O_3_ nanoparticles are uniformly coated with biomass-derived carbon from bagasse—an abundant agricultural residue. Crystalline cellulose extracted from bagasse serves as a green carbon precursor, enabling the formation of a core–shell nanostructure *via* a simple sol–gel and pyrolysis route. With an optimized Fe_2_O_3_ : C weight ratio of 8 : 2 and a polyacrylic acid binder, the electrode delivers a high reversible capacity of 1893 mA h g^−1^ after 100 cycles at 0.1 A g^−1^, retaining 1553 mA h g^−1^ after 350 cycles at 0.5 A g^−1^, and exhibits excellent rate capability up to 3 A g^−1^, outperforming many previously reported Fe_2_O_3_-based anodes. This superior performance arises from the synergistic effects of Fe_2_O_3_ and the conductive carbon coating, which enhance electron transport, buffer volume expansion, and stabilize the solid electrolyte interphase. This study demonstrates the potential of bagasse valorization for sustainable energy storage and offers a scalable route to high-capacity, long-life LIB anodes, paving the way for eco-friendly and cost-effective electrode production for large-scale applications.

## Abstract 2

Bagasse-derived cellulose enabled green synthesis of Fe_2_O_3_@C nanocomposites showing excellent lithium-ion battery performance with an optimized Fe_2_O_3_ : C ratio (8 : 2) and eco-friendly PAA binder.

## Graphical abstract

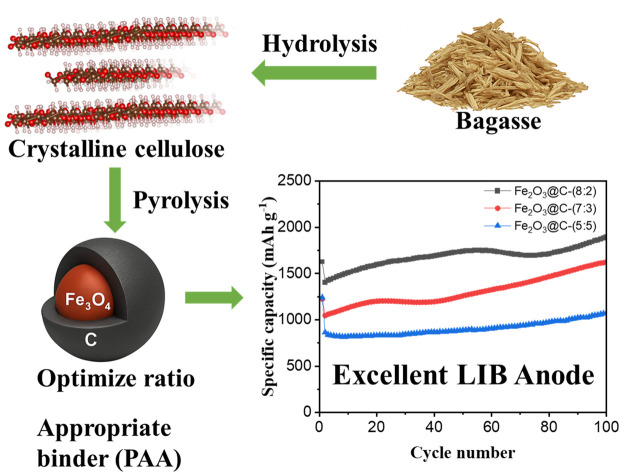

## 1 Introduction

The increasing demand for high-performance energy-storage systems has stimulated extensive research on various types of rechargeable batteries, including lithium-ion batteries (LIBs),^1^ lithium-metal batteries,^2^ lithium/fluorinated carbon batteries,^3^ aqueous batteries,^4^ zinc-based batteries,^5,6^ lithium–oxygen (Li–O_2_) batteries^7,8^ and lithium–carbon dioxide (Li–CO_2_) batteries.^9^ These technologies are essential for powering modern electronic devices such as smartphones, laptops, and wearable technologies. Among them, LIBs have gained prominence because of their high energy density, long cycle life, and stable voltage operation.^10,11^ However, the limited performance of traditional graphite anodes, with a theoretical capacity of only 372 mA h g^−1^, has driven research on alternative anode materials with higher capacity and superior rate capability.^12^ In recent years, transition-metal oxides (TMOs) have emerged as promising candidates for next-generation anode materials in LIBs owing to their high theoretical capacities, diverse oxidation states, and abundance. Compared with conventional graphite anodes, many TMOs, such as Fe_2_O_3_, Co_3_O_4_, and MnO_2_, offer higher capacities exceeding 700 mA h g^−1^. These materials undergo conversion reactions with Li ions, enabling improved energy storage. However, their practical application is limited by issues such as poor electrical conductivity and volume expansion during cycling. To overcome these drawbacks, recent studies have focused on nanostructuring, carbon compositing, and surface modification to enhance the electrochemical performance of TMOs, thereby paving the way for their integration into high-performance LIB systems.^13^

Among TMOs, Fe_2_O_3_ has attracted significant attention owing to its high theoretical capacity (∼1005 mA h g^−1^), low cost, environmental benignity, and natural abundance.^14,15^ Fe_2_O_3_ stores Li *via* a conversion reaction, enabling high-energy storage. However, it suffers from major drawbacks such as large volume expansion during cycling (>200%), poor intrinsic electrical conductivity, and instability of the solid electrolyte interphase (SEI), which lead to pulverization of the active materials and rapid capacity fading.^16–20^ To mitigate these issues, considerable effort has been directed toward the development of Fe_2_O_3_-carbon composites that buffer mechanical stress, enhance electrical conductivity, and stabilize the SEI layer.^21,22^

Among various carbon materials, such as graphene, carbon nanotubes, and soft and hard carbon, biomass-derived carbon is particularly appealing owing to its sustainability, cost-effectiveness, and high structural tunability.^1–3,6,23–26^ Bagasse—a major agricultural byproduct in tropical countries such as Vietnam—is particularly attractive as a green carbon precursor. It consists of approximately 40–50 wt% cellulose, 20–30 wt% hemicellulose, and 18–24 wt% lignin, featuring a carbon-rich composition.^25,27^ Through pretreatment and pyrolysis, crystalline cellulose (CC) can be extracted from bagasse and converted into conductive, porous, sheet-like carbon with a large surface area and mechanical integrity.^28^ These features make bagasse-derived carbon highly suitable for forming core–shell structures with Fe_2_O_3_ nanoparticles, as it effectively buffers volume expansion and improves electrochemical kinetics.^16^

In addition to the material composition, electrode binder chemistry plays a crucial role. While polyvinylidene fluoride (PVDF) is the industry standard, its weak van der Waals interactions with active particles and need for toxic solvents such as *N*-methyl-2-pyrrolidone (NMP) reduce mechanical stability and sustainability. In contrast, water-soluble binders such as polyacrylic acid (PAA) can form strong hydrogen bonds with carbon and oxide surfaces, enhancing adhesion, flexibility, and SEI stability.^23,29,30^ Few studies have comprehensively investigated the integration of Fe_2_O_3_ with sugarcane bagasse (SCB)-derived carbon, particularly in combination with binder engineering. Key challenges remain in optimizing the weight ratio of Fe_2_O_3_ to C, controlling particle size, ensuring uniform carbon coating, and improving long-term cycling stability at practical current densities.

In this work, we report the synthesis of Fe_2_O_3_@C nanocomposites using Fe(NO_3_)_3_ and CC derived from SCB *via* two facile steps, including sol–gel and pyrolysis methods. Three composite ratios (8 : 2, 7 : 3, and 5 : 5) were tested. The effects of the carbon content and binder type (PVDF and PAA) on the structural and electrochemical properties of the resulting materials were systematically studied. Our results demonstrate that SCB-derived carbon facilitates the formation of a robust core–shell architecture, resulting in enhanced Li storage capacity, excellent rate capability, and long-term cycling stability. Thus, it is a promising candidate for next-generation LIB anodes.

Although Fe_2_O_3_@C composites have been widely investigated as anode materials, most previous studies relied on synthetic carbon sources such as graphene, CNTs, or polymer-derived carbon, *etc.*, and often involving complicated synthesis and high costs. In contrast, this work introduces a sustainable and facile strategy by using sugarcane bagasse, a low-cost agricultural byproduct, as the carbon precursor through a simple sol–gel and pyrolysis route. Moreover, we systematically optimized the Fe_2_O_3_ : C ratio and employed a water-soluble PAA binder instead of PVDF, resulting in improved electrode adhesion and structural stability. These strategies yield remarkable electrochemical performance (1893 mA h g^−1^ after 100 cycles at 0.1 A g^−1^ and 1553 mA h g^−1^ after 350 cycles at 0.5 A g^−1^), surpassing most reported Fe_2_O_3_@C works. The superior behavior originates from the synergistic effects of the uniform biomass-derived carbon coating and the flexible PAA binder, which together enhance conductivity, buffer volume expansion, and stabilize the SEI layer. Therefore, this study provides both a sustainable materials concept and an improved electrochemical mechanism for achieving long-life and high-capacity Fe_2_O_3_-based lithium-ion battery anodes.

## 2 Experimental section

### 2.1 Materials

Bagasse from Vietnam was collected, washed, dried, and ground into powder. Iron(iii) nitrate nonahydrate (Fe(NO_3_)_3_·9H_2_O, 98%, Sigma-Aldrich), ethylene glycol (EG, 99%, JHD GHTECH), oxalic acid dihydrate (H_2_C_2_O_4_·2H_2_O, 99.5%, JHD GHTECH), acetic acid (C_2_H_4_O_2_, 99.5%, JHD GHTECH), hydrogen peroxide (H_2_O_2_, 30%, Xilong), sulfuric acid (H_2_SO_4_, 98%, Vietnam), and sodium hydroxide (NaOH, 98%, Vietnam) were of analytical grade and used without further purification.

### 2.2 Extraction of crystalline cellulose (CC) from bagasse

The powdered bagasse was first bleached *via* sequential treatment with acetic acid (80 wt%, 80 °C, 2 h) and hydrogen peroxide (20 wt%, 80 °C, 2 h) to eliminate lignin and hemicellulose. The resulting cellulose pulp was subsequently washed with deionized (DI) water and hydrolyzed with sulfuric acid (60 wt%) under reflux at 60 °C for 2 h to remove amorphous regions. The final product was filtered, washed repeatedly to achieve a neutral pH, and freeze-dried at −40 °C for 8 h to obtain CC powder.

### 2.3 Synthesis of Fe_2_O_3_@C composites

First, Fe(NO_3_)_3_·9H_2_O (1 M) was dissolved in a mixture of oxalic acid (1 M) and ethylene glycol (1 M) at a volumetric ratio of 1 : 1 to prepare the Fe precursor solution. Subsequently, the extracted CC was added and dispersed under stirring to obtain a uniform suspension. Three samples of Fe_2_O_3_@C composites with different weight ratios of Fe_2_O_3_ to carbon (8 : 2, 7 : 3, and 5 : 5) were prepared by mixing the calculated amounts of Fe precursor and cellulose suspension. The resulting mixture was ultrasonicated at 300 W for 30 min to ensure homogeneous dispersion, followed by aging at 90 °C under continuous stirring (600 rpm) until gelation occurred. The gel was dried at 100 °C overnight to remove residual solvent, followed by pyrolysis in a tubular furnace under an N_2_ atmosphere at 450 °C for 6 h. This thermal treatment resulted in the formation of an Fe_2_O_3_@C composite with a core–shell structure, where Fe_2_O_3_ nanoparticles were uniformly coated by carbon derived from bagasse. For comparison, pure-Fe_2_O_3_ powder was prepared using the same procedure without adding CC solution, and the bagasse-derived carbon was obtained *via* thermal treatment of CC powder at 450 °C for 6 h.

### 2.4 Electrode preparation and electrochemical characterization

The working electrode was prepared by mixing Fe_2_O_3_@C, Super P carbon black, and binder (either PVDF or PAA) in a weight ratio of 8 : 1 : 1. NMP was used as the solvent for the PVDF-based electrodes, and DI water was used for the PAA-based electrodes. The slurry was cast onto Cu foil using a doctor blade and dried at 80 °C under vacuum for 12 h. CR2032-type coin cells were assembled in an Ar-filled glove box using Li foil as the counter electrode, a polypropylene membrane as the separator, and 1 M LiPF_6_ in ethylene carbonate: diethylene carbonate (1 : 1 v/v) as the electrolyte. The typical active material and electrolyte loading was 1.0–1.2 mg cm^−2^ and 120 µL for each cell. Galvanostatic charge/discharge (GCD) tests were conducted using a battery tester in the voltage range of 0.01–3.0 V (*vs.* Li^+^/Li). Cyclic voltammetry (CV) and electrochemical impedance spectroscopy (EIS) were performed to evaluate the redox behavior and charge-transfer resistance.

### 2.5 Material characterization

The structural, morphological, and surface properties of the synthesized Fe_2_O_3_@C composites were characterized using various techniques. X-ray diffraction (XRD) analysis was performed using a Bruker D8 Advance diffractometer with Cu Kα radiation (*λ* = 1.5406 Å), operated at 40 kV and 40 mA over the 2*θ* range of 10–80°. Scanning electron microscopy (SEM) (Hitachi S-4800) and high-resolution transmission electron microscopy (HRTEM) (JEOL JEM-2100) were employed to examine the surface morphology, particle size, and distribution of Fe_2_O_3_ within the carbon matrix. Energy-dispersive X-ray spectroscopy (EDS) coupled with SEM was used for elemental mapping to confirm the spatial distribution of Fe, O, and C in the composites. N_2_ adsorption–desorption measurements were performed at 77 K using a Micromeritics ASAP 2020 instrument. The Brunauer–Emmett–Teller (BET) method was used to calculate the specific surface area, and the Barrett–Joyner–Halenda (BJH) model was used to determine the pore size distribution from the desorption branch of the isotherm. The chemical state study of etch elements were performed high-performance X-ray photoelectron spectroscopy: HP-XPS (BS101), K-ALPHA+, Thermo Fisher Scientific Inc. (UK) using monochromated Al Kα X-ray source (*hν* = 1486.6 eV, power = 12 kV, 72 W) at a spot size of 400 µm in diameter with charge compensation using two flood gun (low energy electron and Ar^+^ ion) at Yeongnam Regional Center of Korea Basic Science Institute (KBSI).

## 3 Results and discussion

**Fig. 1:**
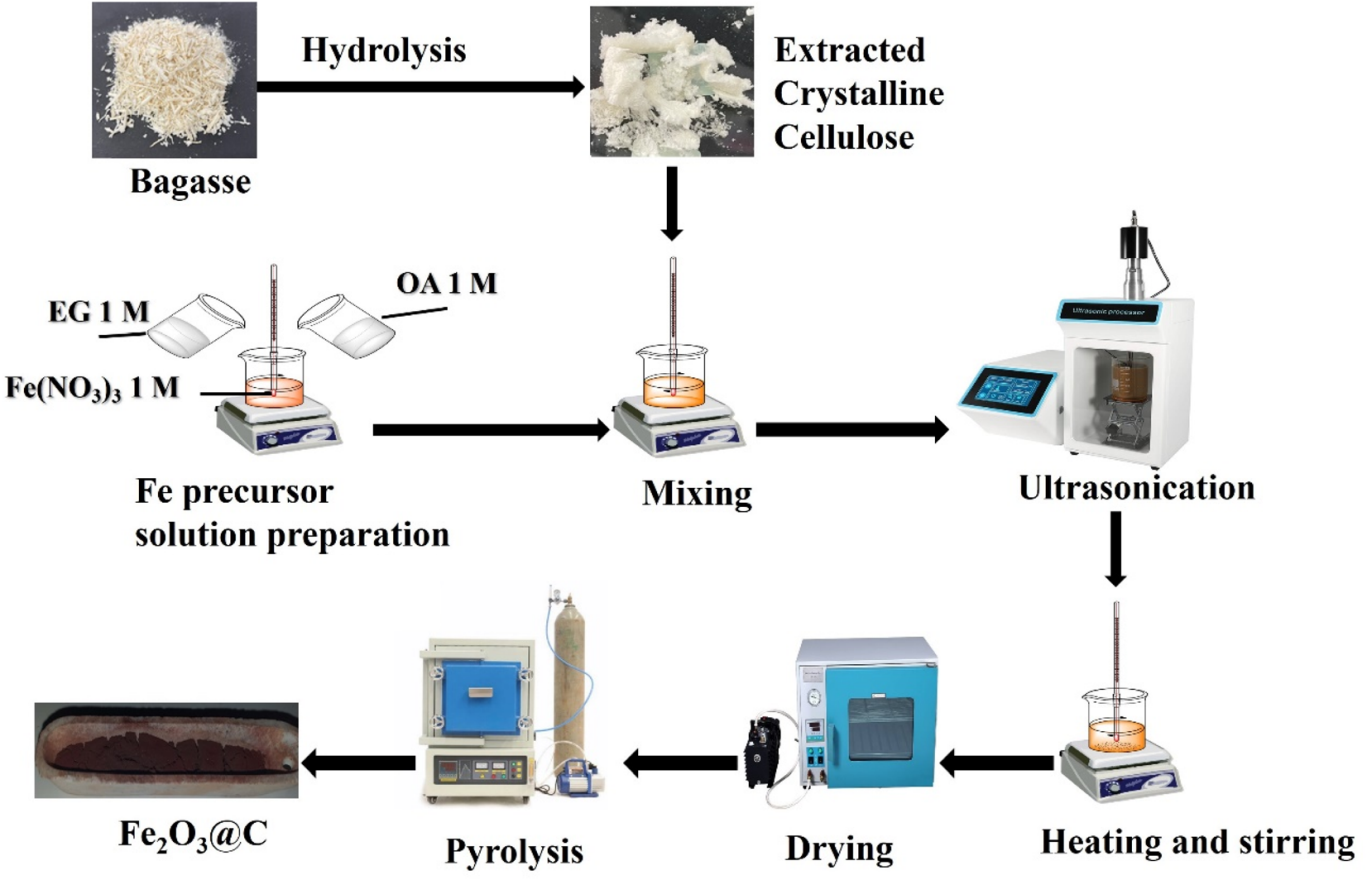
Scheme of synthesis of core–shell Fe_2_O_3_@C nanoparticles.

The synthesis process of the Fe_2_O_3_@C nanoparticles is illustrated in Fig. 1 and described in the Experimental Section. The formation of the Fe_2_O_3_@C composite is governed by a sol–gel process during the mixing and aging steps. Initially, Fe^3+^ ions from Fe(NO_3_)_3_·9H_2_O interact with oxalic acid to form Fe–oxalate complexes, preventing premature precipitation of Fe(OH)_3_. EG functions as a polyol, promoting esterification and polycondensation reactions with oxalic acid. Heating at 90 °C forms an organic polymeric network that traps Fe^3+^ species uniformly within the gel matrix. This gelation step ensures the homogeneous distribution of the Fe precursor and cellulose within the network. Upon drying and pyrolysis under N_2_, the carbon-phase component derived from CC is formed *via* pyrolysis (eqn (1)),^28,31^ while the coordinated Fe^3+^ species is converted to Fe_2_O_3_ nanoparticles *via* two steps, as given by eqn (2) and (3), resulting in a core–shell structure where carbon derived from cellulose encapsulates Fe_2_O_3_ nanoparticles.^32^

1 Cellulose → C (s) + CO (g) + H_2_O (l)

2 Fe^3+^ + 3OH^−^ → Fe(OH)_3_ (s)

3 2Fe(OH)_3_ (s) → Fe_2_O_3_ (s) + 3H_2_O (g)

**Fig. 2:**
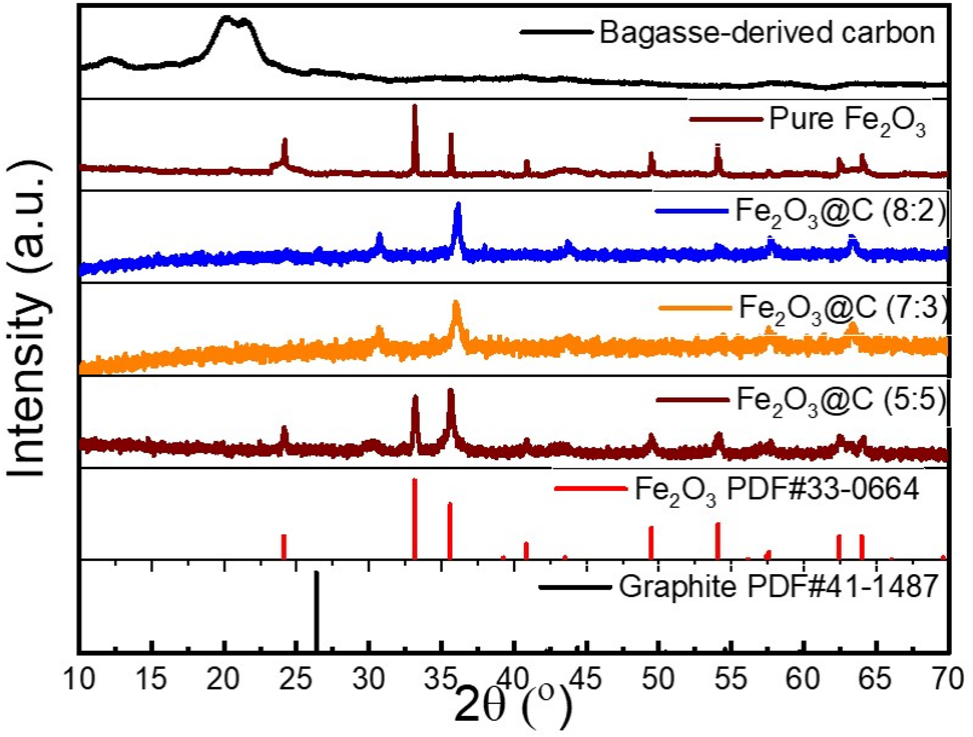
XRD patterns of Fe_2_O_3_@C with various ratios (5 : 5, 7 : 3, and 8 : 2), pure Fe_2_O_3_, and bagasse-derived carbon.

Fig. 2 shows the XRD patterns of the bagasse-derived carbon, pure Fe_2_O_3_, and the Fe_2_O_3_@C composites (8 : 2, 7 : 3, and 5 : 5). The Fe_2_O_3_-based samples exhibited sharp diffraction peaks at 2*θ* ≈ 24.1°, 33.2°, 35.6°, 40.9°, 49.5°, 54.1°, 57.7°, 62.4°, and 64.0°, corresponding to the (012), (104), (110), (113), (024), (116), (018), (214), and (300) crystal planes of rhombohedral hematite Fe_2_O_3_ (JCPDS no. 33-0664), respectively. These results confirmed the formation of the high-crystallinity phase α-Fe_2_O_3_. In contrast, the bagasse-derived carbon exhibited a broadened peak at 2*θ* ≈ 22°, characteristic of amorphous carbon derived from biomass pyrolysis.^33,34^ For comparison, the (002) reflection of graphitic carbon (PDF#41-1487) is typically observed at 2*θ* ≈ 26.2°. The absence of this distinct peak in our samples confirms that the bagasse-derived carbon remains largely amorphous. In addition, all Fe_2_O_3_@C samples exhibited only a faint hump at 2*θ* ≈ 20–25°, corresponding to cellulose-derived carbon, which remained predominantly amorphous after pyrolysis at 450 °C under N_2_. This weak feature was obscured by the sharp diffraction peaks of Fe_2_O_3_. With increasing Fe_2_O_3_ crystallinity, the strong diffraction of Fe_2_O_3_ dominated the diffractogram, effectively masking the carbon signal. Moreover, a new weak peak at ∼31° appeared, which can be assigned to the (220) plane of a spinel iron oxide (γ-Fe_2_O_3_ or Fe_3_O_4_), formed *via* partial thermal reduction of Fe(iii) species in the carbonaceous matrix during thermal treatment under N_2_ at 450 °C.^35^ Among the composite samples, Fe_2_O_3_@C-(8 : 2) exhibited the most intense and well-defined diffraction peaks of carbon, suggesting that the moderate carbon content allowed optimal crystallite growth. Fe_2_O_3_@C-(7 : 3) exhibited slightly reduced peak intensities, and Fe_2_O_3_@C-(5 : 5) exhibited broader peaks with reduced intensities, indicating a tendency to transform into amorphous carbon with an increase in carbon content. No secondary phases or impurities (such as Fe_3_O_4_ or Fe) were detected, confirming the high purity of the Fe_2_O_3_ materials. This structural integrity is essential for achieving stable conversion reactions during lithiation/delithiation in LIBs.

**Fig. 3:**
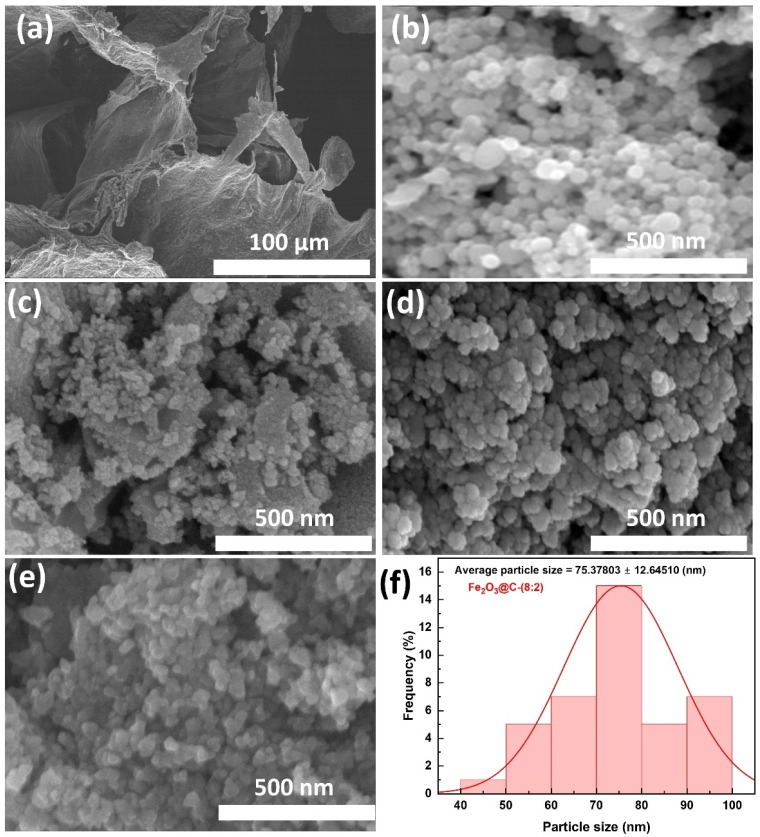
SEM images of (a) bagasse-derived carbon, (b) pure Fe_2_O_3_, (c) Fe_2_O_3_@C-(5 : 5), (d) Fe_2_O_3_@C-(7 : 3), and (e) Fe_2_O_3_@C-(8 : 2); (f) particle size distribution of Fe_2_O_3_@C-(8 : 2).

The surface morphologies of the as-prepared samples were examined using SEM, as shown in Fig. 3. These images reveal the structural differences among the bagasse-derived carbon, pure Fe_2_O_3_, and Fe_2_O_3_@C nanocomposites synthesized at various Fe_2_O_3_ : C mass ratios. Fig. 3a shows that the carbon obtained from bagasse pyrolysis has a thin, wrinkled, and layered sheet-like morphology in micro size, which is typical for biomass-derived carbon materials. This porous structure increases the surface area and provides a network for dispersing and covering smaller particles.^36^ In contrast, the pure-Fe_2_O_3_ sample (Fig. 3b) exhibited dense and aggregated spherical nanoparticles with poor dispersion. This agglomeration is attributed to strong magnetic–dipole interactions and surface energy, which can lead to poor electrochemical performance owing to hindered Li^+^ diffusion and limited active sites.^37^

**Fig. 4:**
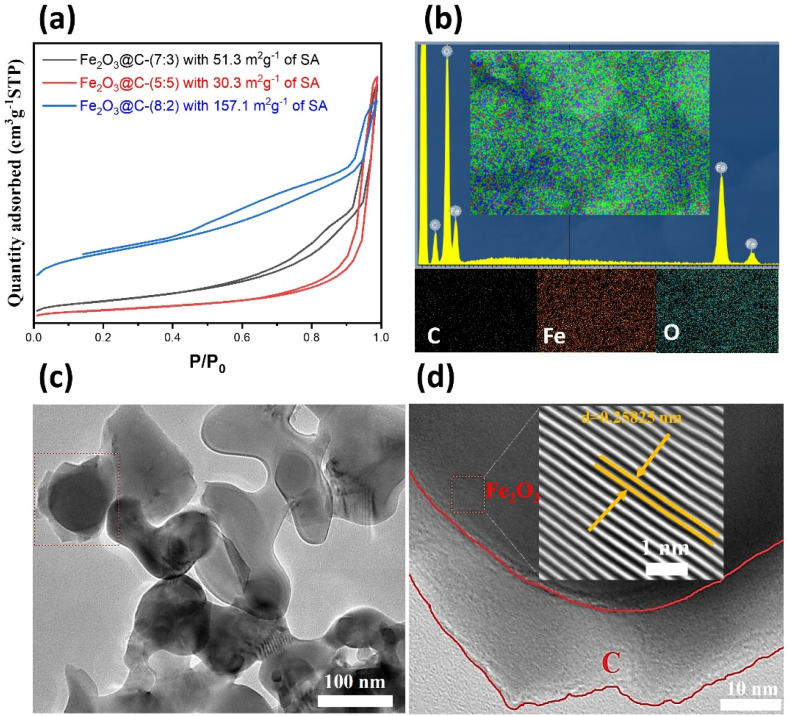
(a) N_2_ adsorption–desorption isotherms (BET surface area) of Fe_2_O_3_@C composites; (b) EDS elemental mapping of Fe_2_O_3_@C-(8 : 2); (c and d) TEM and HRTEM images of Fe_2_O_3_@C-(8 : 2), showing a core–shell Fe_2_O_3_–C architecture.

The introduction of carbon significantly altered the morphologies of the composites. Fe_2_O_3_@C-(5 : 5) (Fig. 3c) exhibited irregularly dispersed particles within a relatively thick carbon matrix. Excess carbon likely led to partial encapsulation of the active material, reducing the accessibility of Fe_2_O_3_ to the electrolyte and potentially hindering electron transfer. Fe_2_O_3_@C-(7 : 3) (Fig. 3d) exhibited moderate dispersion of Fe_2_O_3_ nanoparticles with reduced agglomeration. The particles appeared to be more evenly distributed within the carbon framework, indicating improved structural integration. Fe_2_O_3_@C-(8 : 2) (Fig. 3e) exhibited the most homogeneous and uniform distribution of Fe_2_O_3_ nanoparticles, which were well-dispersed within a thin carbon matrix, forming a favorable core–shell configuration, as confirmed by the transmission electron microscopy (TEM) images in Fig. 4c and d. This optimized morphology is expected to facilitate ion and electron transport and buffer mechanical stress during cycling, leading to enhanced electrochemical performance.^16,29^ In addition, the particle size distributions of the Fe_2_O_3_@C samples were examined (Fig. 3f and S1), revealing the smallest average particle diameter (∼75.38 ± 12.65 nm) for the Fe_2_O_3_@C-(8 : 2) sample, compared to ∼84.43 ± 7.73 nm for Fe_2_O_3_@C-(7 : 3) and ∼100.04 ± 14.28 nm for Fe_2_O_3_@C-(5 : 5). The relatively narrow size distribution of all samples indicates effective control over nucleation and growth during synthesis, which is crucial for achieving stable cycling performance and high rate capability.^14^ Notably, the smallest particle size of Fe_2_O_3_@C-(8 : 2) suggests that its thinner carbon layer—resulting from the lower carbon content—may facilitate faster charge transfer by reducing the interfacial resistance during charge/discharge processes.

To further investigate the nanostructure and composition of the synthesized Fe_2_O_3_@C composites, BET surface area analysis, EDS mapping, and HRTEM were employed. The N_2_ adsorption–desorption isotherms (Fig. 4a) indicated that Fe_2_O_3_@C-(8 : 2) had a significantly larger specific surface area (157.1 m^2^ g^−1^) than Fe_2_O_3_@C-(5 : 5) (30.3 m^2^ g^−1^) and Fe_2_O_3_@C-(7 : 3) (51.3 m^2^ g^−1^). This is attributed to the optimized carbon content, which prevents Fe_2_O_3_ nanoparticle agglomeration and forms a porous carbon matrix. A larger surface area provides more active sites for Li storage and enhances the electrolyte infiltration, leading to improved electrochemical kinetics.^21^ In addition, Fig. 4b shows the EDS elemental mapping of Fe_2_O_3_@C-(8 : 2), which confirms the uniform distribution of Fe, O, and C throughout the composite. The even dispersion of Fe and O indicates homogeneous formation of Fe_2_O_3_ nanograins, while the surrounding carbon matrix ensures continuous electrical contact. This compositional uniformity plays a key role in maintaining structural stability during repeated charge/discharge cycles.^38^ Moreover, the TEM image in Fig. 4c reveals discrete Fe_2_O_3_ nanoparticles embedded within a thin carbon matrix, consistent with a well-dispersed core–shell architecture. The particles were quasi-spherical and exhibited minimal aggregation, which was consistent with the SEM and BET observations. Furthermore, an HRTEM image (Fig. 4d) showed that this material consisted of Fe_2_O_3_ particles encapsulated in carbon. The spacing between the lattice planes was determined to be approximately 0.258 nm, corresponding to the (110) plane of the Fe_2_O_3_ material. The core–shell structure comprised a crystalline Fe_2_O_3_ core surrounded by a carbon shell with a thickness of approximately 10 nm. The carbon layer is continuous and wraps the Fe_2_O_3_ core, which helps accommodate volume expansion, maintain electrical connectivity, and suppress the pulverization effect common in TMO anodes.^18^

**Fig. 5:**
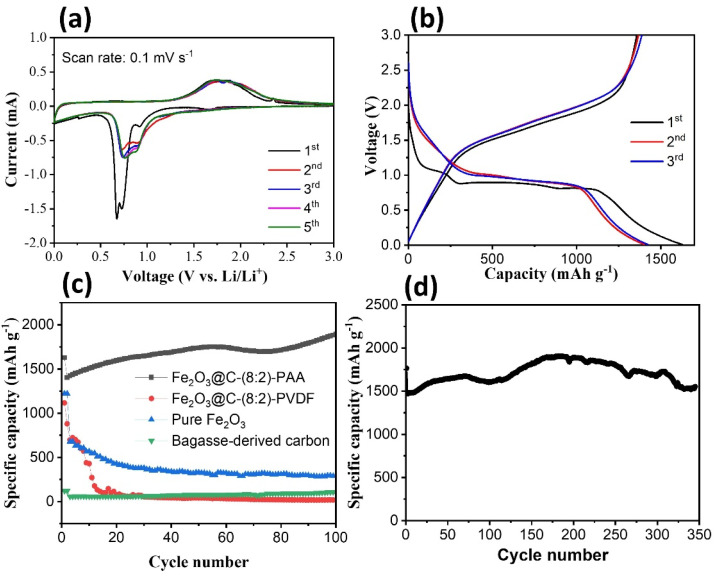
(a) CV curves at 0.1 mV s^−1^; (b) charge/discharge profiles of the Fe_2_O_3_@C-8 : 2 composite at 0.1 A g^−1^ during the first three cycles; (c) cycling performance at 0.1 A g^−1^ of different Fe_2_O_3_@C composites; (d) long-term cycling performance of Fe_2_O_3_@C-8 : 2 at 0.5 A g^−1^.

The CV and GCD tests (Fig. 5a and b) were performed to evaluate the electrochemical behavior of the Fe_2_O_3_@C composites. The CV curves of Fe_2_O_3_@C were recorded at a scan rate of 0.1 mV s^−1^ in the voltage window of 0.01–3.0 V *vs.* Li/Li^+^ (Fig. 5a). In the first cathode scan, the peaks at ∼0.92, ∼0.72, and ∼0.67 V are attributed to the insertion of ions Li^+^ into the structure of the Fe_2_O_3_ material (eqn (4)), the reduction of Fe(iii) to Fe(0) (eqn (5)), and the development of the SEI layer, respectively. The disappearance of the peak at ∼0.67 V in subsequent cycles indicated that the SEI formation was irreversible. The cathode peaks in the subsequent cycles exhibited a shift in position compared to the first cycle, specifically at ∼0.9 and ∼0.75 V. This phenomenon may be related to the formation of an SEI layer during the first cycle. In contrast, the anode peak exhibited consistent oxidation-peak positions in all five cycles. The broad peak appearing at ∼1.75 V may represent the transition from Fe^0^ to Fe^3+^.^39–41^ In addition, the overlap of the CV curves was almost identical after the first discharge cycle, which suggests the good reversible redox behavior of the Fe_2_O_3_@C electrode. The electrochemical reaction mechanism of the Fe_2_O_3_@C composite can be summarized as follows:

4 Fe_2_O_3_ + *x*Li^+^ + *x*e^−^ ↔ Li_*x*_Fe_2_O_3_

5 Li_*x*_Fe_2_O_3_ + (6 − *x*)Li^+^ + (6 − *x*)e^−^ ↔ 2Fe +3Li_2_O

Fig. 5b presents the voltage profile of the Fe_2_O_3_@C electrode during the first three cycles at a current density of 0.1 A g^−1^, which exhibits characteristic plateaus, consistent with the CV results. The first discharge capacity exceeds 1627 mA h g^−1^ but with irreversible capacity loss due to SEI formation and electrolyte decomposition, corresponding to a coulombic efficiency of ∼83%.^42^ The reversible capacity stabilizes at ∼1406 mA h g^−1^ from the second cycle onward, and the coulombic efficiency increases to ∼98% for the second and third cycles. The relatively flat discharge plateau near ∼0.9, ∼0.8, and ∼0.6 V and the charge plateau near ∼1.75 V confirm the conversion-type reaction mechanism revealed by CV analysis. Voltage hysteresis between charge and discharge is typical for Fe_2_O_3_ anodes and can be mitigated by the carbon matrix, which enhances conductivity and structural integrity. The aforementioned findings indicated the good reversible discharge capability of the Fe_2_O_3_@C electrode.

The cycling performance of the Fe_2_O_3_@C electrode at a current density of 0.1 A g^−1^ was evaluated in comparison with pure Fe_2_O_3_ and bagasse-derived carbon, along with the effect of the binder on the Fe_2_O_3_@C electrode (Fig. 5c). The electrodes prepared with the PVDF binder for Fe_2_O_3_@C, pure Fe_2_O_3_, and bagasse-derived carbon exhibited a sharp decline in capacity after just a few cycles. Among them, the carbon electrode exhibited a low capacity of ∼121 mA h g^−1^ at the 1st cycle, and its capacity remained at ∼108 mA h g^−1^ after the 100th cycle. The pure-Fe_2_O_3_ electrode exhibited the highest specific capacity at the first cycle (∼1221 mA h g^−1^), compared to ∼1116 mA h g^−1^ for Fe_2_O_3_@C; however, this value was reduced to 632 mA h g^−1^ after five cycles, which was lower than the capacity of Fe_2_O_3_@C (701 mA h g^−1^). The significant capacity loss for the pure-Fe_2_O_3_ electrode could be due to the large volume expansion of the electrode material after repeated charge/discharge cycles, which destroyed the structure of the material. Moreover, the specific capacity of Fe_2_O_3_@C using the PVDF binder continued to decrease significantly over 100 cycles, reaching ∼17.5^−1^ mA h g^−1^, primarily because of the mechanical and interfacial limitations of the PVDF binder when paired with conversion-type anode materials such as Fe_2_O_3_. First, PVDF lacks sufficient elasticity to accommodate large volume variations of Fe_2_O_3_ during lithiation/delithiation (>200%) in conversion-type anodes, leading to cracking and pulverization of the electrode and loss of electrical contact between active-material particles and the current collector.^43^ Second, PVDF is nonpolar and chemically inert, offering weak interfacial binding with metal-oxide surfaces such as Fe_2_O_3_. This contributes to poor adhesion and structural instability during repeated cycling, resulting in Fe_2_O_3_ particles being detached from the electrode matrix and becoming electrochemically inactive owing to poor electronic connectivity. In contrast, the Fe_2_O_3_@C electrode using the PAA binder exhibited a specific capacity of 1627 mA h g^−1^ at the first cycle, and this value tended to increase during the discharge process, reaching 1893 mA h g^−1^ at the 100th cycle. PAA forms strong hydrogen bonds with oxide surfaces, which improves the mechanical integrity and adhesion of the electrodes compared to PVDF.^44^ Moreover, PAA exhibits elastic and ductile mechanical behavior, allowing it to maintain electrode integrity under significant morphological changes, whereas PVDF is relatively brittle and less resilient.^45,46^ Furthermore, PAA interacts more effectively with conductive additives, such as carbon, facilitating the formation of a continuous conductive network. This enhancement promotes charge transfer and electronic conductivity throughout the electrode, which is crucial for Fe_2_O_3_ because of its intrinsically low conductivity. When the charge/discharge process was performed at a high current density of 0.5 A g^−1^ (Fig. 5d), the Fe_2_O_3_@C-(8 : 2) electrode still exhibited a remarkable capacity of ∼1552 mA h g^−1^ after 350 cycles. This stability stems from the core–shell structure and optimal carbon content (20 wt%), which effectively buffer the large volume changes of Fe_2_O_3_ and preserve the structural integrity of the electrode.^47,48^

**Fig. 6:**
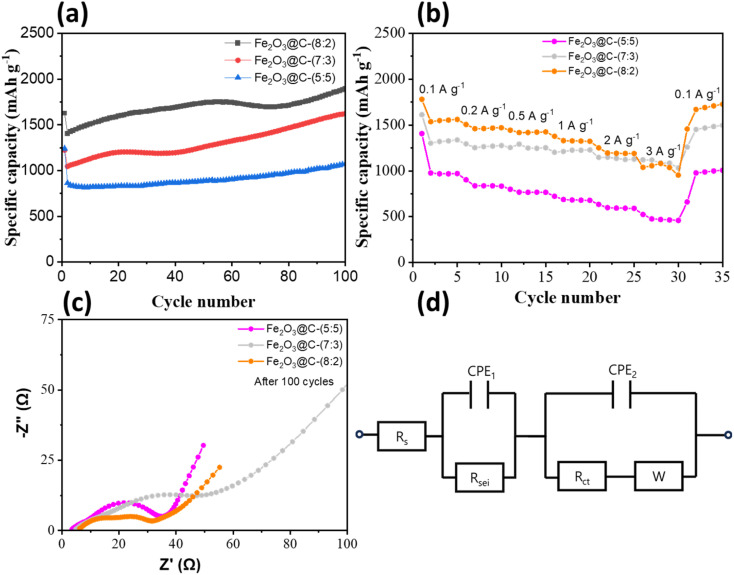
(a) Cycling performance at a current density of 0.1 A g^−1^ of Fe_2_O_3_@C composites with different Fe_2_O_3_-to-carbon mass ratios; (b) rate capability of Fe_2_O_3_@C composites; (c) EIS plots of Fe_2_O_3_@C electrodes after the cycling test; (d) equivalent circuit model.

Fig. 6a demonstrates the impact of the Fe_2_O_3_ : C mass ratio on the cycling stability of the composite electrodes. The Fe_2_O_3_@C-(8 : 2) electrode exhibited a specific capacity of 1627 mA h g^−1^ at the first cycle, and this value tended to increase during the discharge process, reaching 1893 mA h g^−1^ at the 100th cycle. Similarly, the specific capacity of the Fe_2_O_3_@C-(7 : 3) and Fe_2_O_3_@C-(5 : 5) electrodes increased to ∼1624 and ∼1070 mA h g^−1^, respectively, after 100 discharge cycles. This enhancement is primarily attributed to the structural integrity and strong interfacial contact between Fe_2_O_3_ and the biomass-derived carbon matrix. In contrast to conventional carbon additives, carbon from bagasse forms a continuous and flexible network that accommodates volume changes, suppresses particle pulverization, and maintains electronic conductivity. The superior performance of Fe_2_O_3_@C-(8 : 2) compared to Fe_2_O_3_@C-(7 : 3) and Fe_2_O_3_@C-(5 : 5) is ascribed to the optimal carbon content, which provides sufficient electronic pathways and structural cushioning without excessively diluting the active-material (Fe_2_O_3_) content. Excess carbon reduces the volumetric energy density and prevents aggregation and cracking during cycling.^16^ The gradual capacity increase during cycling observed in Fig. 5c and 6a can be ascribed to the electrode activation process, including gradual wetting and penetration of the electrolyte; the reversible formation of polymeric gel-like films, which contribute pseudocapacitive capacity; enhanced reversibility of the conversion reaction due to structural rearrangements of Fe_2_O_3_@C nanocomposite and increased electronic conductivity from the carbon coating. This behavior is intrinsic to Fe_2_O_3_-based anodes and is not caused by electrolyte consumption, as confirmed by the reduced R_ct_ observed in the EIS.^13,16,17,37,49^

Furthermore, as shown in Fig. 6b, the rate capability of the composite electrodes is evaluated at increasing current densities ranging from 0.1 to 3 A g^−1^. Owing to diffusion limitations, the capacity of the Fe_2_O_3_@C-(8 : 2) electrode decreased gradually from 1780 mA h g^−1^ to 1506, 1444, 1376, 1251, and 1038 mA h g^−1^ as the current increased from 0.1 A g^−1^ to 0.2, 0.5, 1, 2, and 3 A g^−1^, respectively, but it quickly recovered to ∼1726 mA h g^−1^ when the current was returned to 0.1 A g^−1^. Although the specific capacities were lower owing to the lower mass of Fe_2_O_3_ in the composite, the rate capability of the samples with ratios of 7 : 3 and 5 : 5 exhibited the same properties (good recovery of capacitance after a series of current density) as that of the (8 : 2) sample. This excellent rate performance of all samples is attributed to (1) Fe_2_O_3_ nanoparticles that shorten Li^+^ diffusion paths; (2) the uniform carbon coating derived from bagasse, which enhances electrical conductivity; and (3) the porous morphology, as confirmed by BET and TEM analysis, which allows electrolyte penetration and efficient ion transport. These results confirm that the core–shell structure of Fe_2_O_3_@C with the bagasse-derived carbon shell not only enhances long-term durability but also provides outstanding rate performance, which is critical for practical LIB applications where both power and stability are essential. This result is superior to those for most other Fe_2_O_3_-carbon composite anodes in previous works, as summarized in Table S1.

EIS was conducted after 100 cycles to gain insights into the interfacial resistance and charge-transfer kinetics of Fe_2_O_3_@C electrodes with different Fe_2_O_3_ : carbon ratios (Fig. 6c). The fitted parameters based on the equivalent circuit (Fig. 6d) are presented in Table S1, including the solution resistance (*R*_s_), SEI resistance (*R*_SEI_), and charge-transfer resistance (*R*_ct_). *R*_s_ increased significantly as the carbon content decreased, rising from 3.2 Ω (Fe_2_O_3_@C-(5 : 5)) to 5.8 Ω (Fe_2_O_3_@C-(7 : 3)) and 6.6 Ω (Fe_2_O_3_@C-(8 : 2)). This minor variation compared with *R*_ct_ suggests that the electrolyte resistance had a negligible influence on performance. In addition, the increase in *R*_s_ compared to fresh cells (Table S2) likely originated from the reduced porosity and additional SEI formation on the Fe_2_O_3_-rich electrodes after cycling.^12,17^ Notably, *R*_ct_ exhibited the opposite trend to that for the fresh cells (Fig. S2). It decreased with a lower carbon content after cycling. The Fe_2_O_3_@C-(8 : 2) electrode exhibited the lowest *R*_ct_ (8.5 Ω) after 100 cycles, whereas the carbon-rich sample (Fe_2_O_3_@C-(5 : 5)) exhibited the highest *R*_ct_ (180.5 Ω). This indicates that Fe_2_O_3_-rich electrodes undergo structural reorganization during cycling, generating finely dispersed metallic Fe and a stable SEI, which enhance electronic conductivity and interfacial kinetics. In contrast, excessive carbon promotes partial electrode densification and unstable SEI growth, increasing the interfacial resistance during cycling.^49,50^ For all the samples, *R*_SEI_ was increased after cycling, reflecting ongoing SEI formation and electrolyte decomposition. This increase was most significant for the Fe_2_O_3_@C-(5 : 5) electrode because of its large carbon surface area, which accelerated SEI thickening. In comparison, the Fe_2_O_3_@C-(8 : 2) electrode developed a thinner and more stable SEI, contributing to improved cycling stability and rate performance. In addition, Fig. S3 shows the morphologies of the Fe_2_O_3_@C-(8 : 2) electrodes before and after cycling. Although SEI was formed after cycling test, the presence of a thin and uniform SEI layer could be evidenced by the low charge-transfer resistance, which ensures efficient Li^+^ transport and stable interfacial contact. In addition, the overall porous structure and homogeneous particle dispersion remained well preserved, indicating that the amorphous carbon shell effectively accommodates the volume expansion and prevents particle agglomeration during repeated lithiation/delithiation processes. Moreover, *ex situ* XPS analysis was further employed to examine the surface chemistry of the Fe_2_O_3_@C-(8 : 2) electrodes before and after cycling (Fig. S4). Compared with the pristine electrode, the cycled sample showed the appearance of metallic Fe, while the main peaks of Fe 2p (∼710–725 eV), O 1s (∼530 eV), and C 1s (∼285 eV) are still clearly observed which can be attributed to Fe_2_O_3_ and carbon species. This indicates the reversible conversion between Fe^3+^ and Fe^0^ during the lithiation/delithiation process, consistent with the conversion mechanism described in eqn (4) and (5). Notably, after cycling, a new F 1s signal appeared, and the C 1s spectrum remained dominantly as C–C, C–O, C=O peaks, while the intensities of the C–O and π–π peaks slightly increased, suggesting the formation of a thin SEI layer composed of LiF, Li_2_CO_3_, and organic carbonate species. The absence of significant peak broadening indicates that the SEI is chemically stable and uniform, whereas the carbon shell retains its structural integrity, ensuring good electrical contact and effective mechanical buffering during repeated lithiation/delithiation cycles, which is consistent with the superior performance shown in Fig. 6a.

## 4 Conclusions

A novel core–shell-structured Fe_2_O_3_@C nanocomposite was synthesized using two facile steps, including sol–gel and pyrolysis processes, with bagasse-derived carbon as the carbon matrix. The effects of different Fe_2_O_3_-to-carbon ratios and polymeric binders (PVDF and PAA) on the electrochemical performance of the anodes were systematically studied. Among the tested ratios, Fe_2_O_3_@C-(8 : 2) exhibited the best performance, delivering a high reversible capacity of 1893 mA h g^−1^ after 100 cycles at 0.1 A g^−1^ and 1553 mA h g^−1^ after 350 cycles at 0.5 A g^−1^, along with excellent rate capability up to 3 A g^−1^. This enhancement was attributed to the optimized carbon coating, reduced charge-transfer resistance, and improved ionic/electronic conductivity. Furthermore, replacing PVDF with a PAA binder significantly improved the long-term stability owing to stronger mechanical adhesion and better interfacial contact. This study highlights the potential of low-cost, sustainable biomass-derived carbon combined with Fe_2_O_3_ for the fabrication of efficient and stable anode materials for next-generation LIBs.

## Author contributions

Quoc Hai Nguyen: project administration, writing, review & editing, methodology. Chanwoo Park: methodology, formal analysis, writing. The Sang Chung: methodology, investigation. Thu Huyen Nguyen Thi: methodology, investigation. To Giang Tran: writing, formal analysis. Jong-Seong Bae: formal analysis. Tuan Loi Nguyen: writing – original draft, supervision. Jaehyun Hur: project administration, funding acquisition, review & editing.

## Conflicts of interest

There are no conflicts to declare.

## Supplementary Material

RA-015-D5RA07487H-s001

## Data Availability

All data supporting the findings of this study are available within the article and its supplementary information (SI). Supplementary information is available. See DOI: https://doi.org/10.1039/d5ra07487h.
