# Supplementary material for: Green carbon from bagasse for uniform coating of Fe2O3 nanoparticles toward high-capacity and long-life lithium-ion battery anodes
Source: RSC Adv. 2025 Nov 13;15(52):44134–44. doi: 10.1039/d5ra07487h (PMC12614163; doi:10.1039/d5ra07487h)
Supplement: RA-015-D5RA07487H-s001 [file RA-015-D5RA07487H-s001.pdf]

Supplementary Information (SI) for RSC Advances.
This journal is © The Royal Society of Chemistry 2025

Supporting Information

Green carbon from bagasse for uniform coating of Fe_2O_3 nanoparticles toward high-capacity and long-life lithium-ion battery anodes

Quoc Hai Nguyen[1*†], Chanwoo Park[2†], The Sang Chung[3], Thu Huyen Nguyen Thi[3], To Giang Tran[4,5], Jong-Seong Bae[6], Tuan Loi Nguyen[7,8*], Jaehyun Hur[2*]

[1]Group of Applied Research in Advanced Materials for Sustainable Development, Faculty of Applied Sciences, Ton Duc Thang University, Ho Chi Minh City, Vietnam. E-mail: nguyenquochai@tdtu.edu.vn

[2]School of Chemical, Biological, and Battery Engineering, Gachon University, Seongnam, Gyeonggi 13120, Republic of Korea. E-mail: chanwoo5061@gachon.ac.kr; jhhur@gachon.ac.kr

[3]Faculty of Applied Sciences, Ton Duc Thang University, Ho Chi Minh City, Vietnam. E-mail: chungthesang.st@tdtu.edu.vn; nguyenthithuhuyen.st@tdtu.edu.vn

[4]Institute of Research and Development, Duy Tan University, Da Nang, Vietnam. E-mail: *trantogiang@duytan.edu.vn*

[5]School of Engineering & Technology, Duy Tan University, Da Nang, Vietnam

[6]Yeongnam Regional Center, Korea Basic Science Institute, Busan 46742, Korea. E-mail: jsbae@kbsi.re.kr

[7]Institute of Fundamental and Applied Sciences, Duy Tan University, Ho Chi Minh City 70000, Vietnam. E-mail: nguyentuanloi@duytan.edu.vn

[8]Faculty of Natural Sciences, Duy Tan University, Da Nang City 50000, Vietnam

*Corresponding authors: nguyenquochai@tdtu.edu.vn; nguyentuanloi@duytan.edu.vn; jhhur@gachon.ac.kr

†Q. H. Nguyen and C. Park contributed equally to this study.

(a)

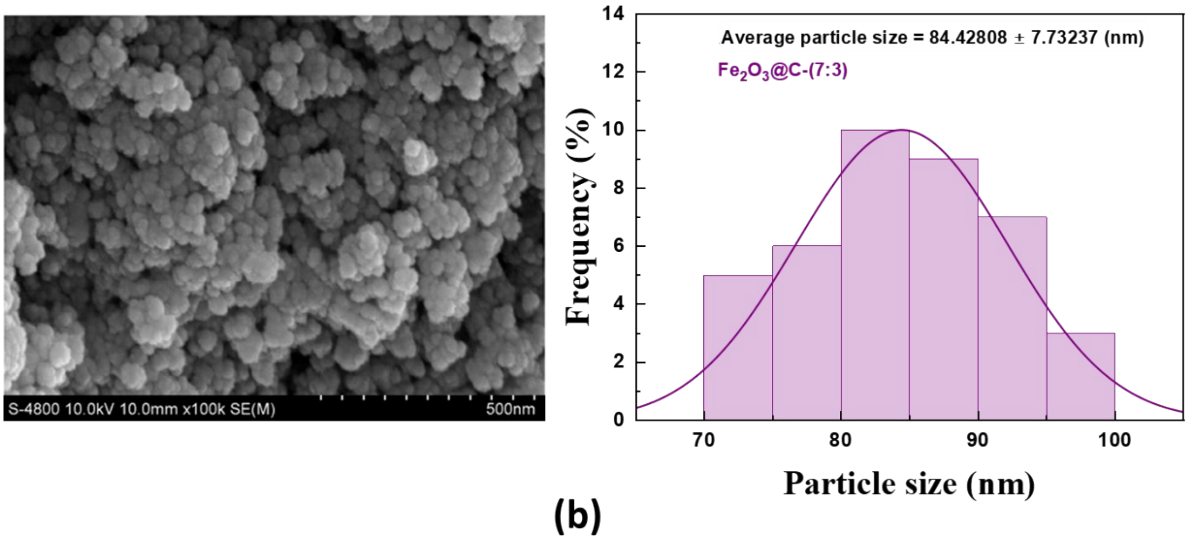

(b)

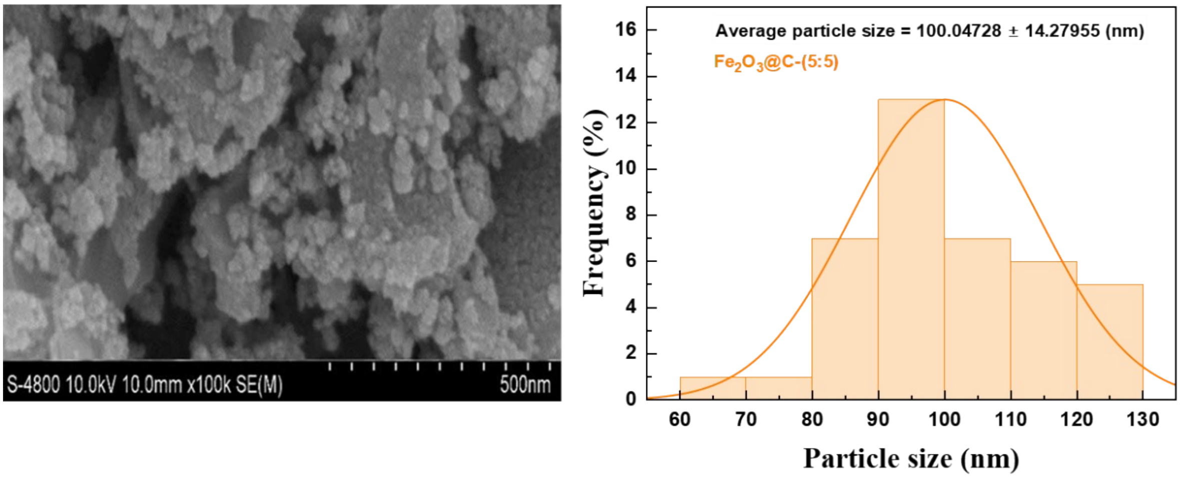

Fig. S1. SEM image and Particle size distribution of (a) Fe_2O_3@C-(7:3) and (b) Fe_2O_3@C-(5:5)

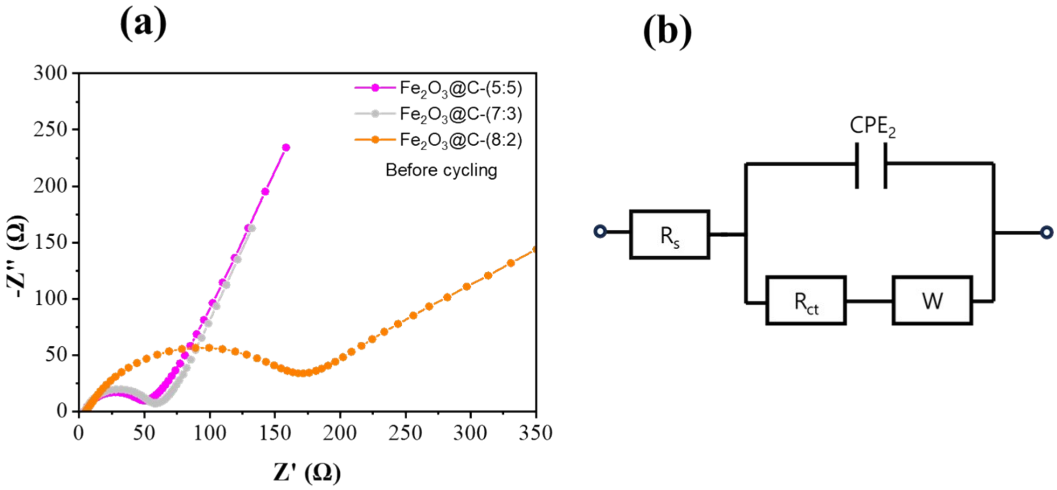

Fig. S2. (a) EIS plots of Fe_2O_3@C electrodes before cycling test; (b) The equivalent electrical circuit model

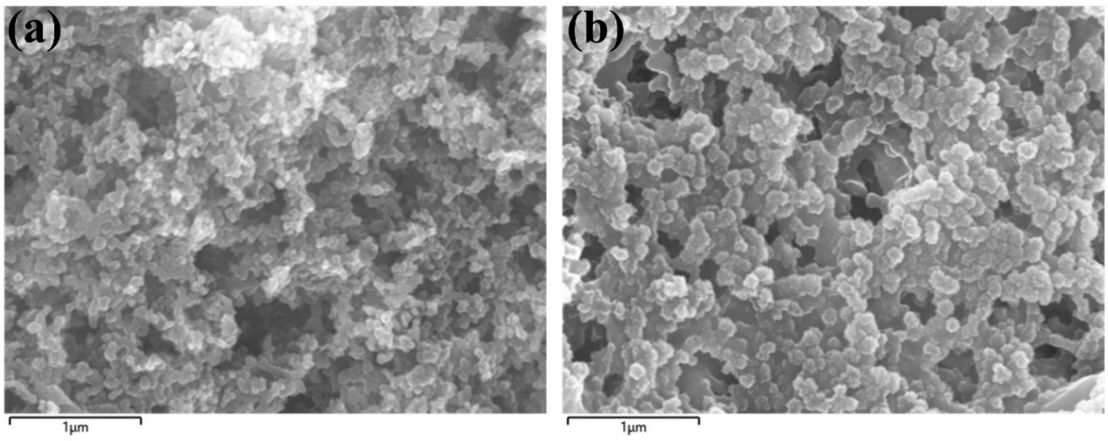

Fig. S3. Ex-situ SEM images of Fe_2O_3@C electrodes (a) before and (b) after cycling (100 cycles).

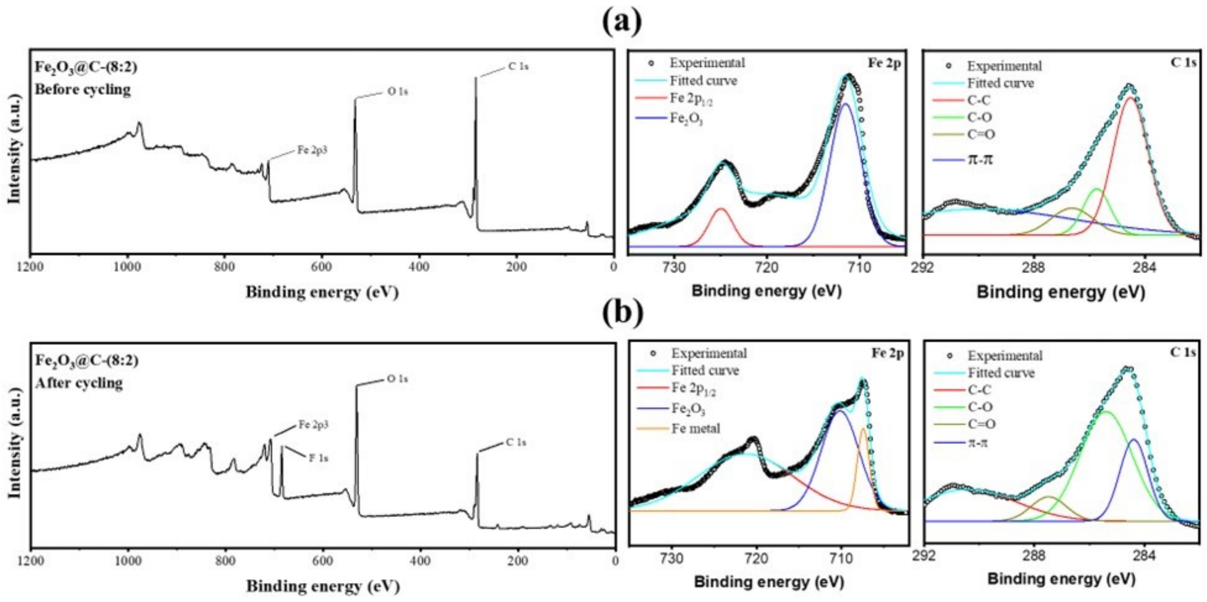

Fig. S4. Ex-situ XPS results of Fe_2O_3@C-(8:2) electrodes (a) before and (b) after cycling (100 cycles).

Table. S1. Comparison of the electrochemical performance in terms of specific capacity and life-span of Fe_2O_3-Carbon composites as anode materials for half-cell LIBs.

**Anode materials**	**Structure**	**Synthesis method**	**Carbon source**	**Maintained capacity (mAh g^{-1})**	**Cycle number**	**Current density**	**Ref.**
Fe_2O_3@C	Uniform carbon shells coating of Fe_2O_3 nanoparticles	Ultrasonication-assisted sol-gel and calcination	Bagasse	1893<br>1553	100<br>350	0.1 A/g<br>0.5 A/g	This work
Fe_2O_3/MAC	One-dimensional Fe_2O_3 nanorods distributed on mesoporous of carbon matrix.	Hydrothermal	Activated carbon	479.9	100	0.1 C	[1]
EMG/Fe_2O_3	Fe_2O_3 nanoparticles attached to the surface and fixed in the layered structure of the EMG	Hydrothermal and calcination	Microcrystalline graphite	1007.05	100	0.1 A/g	[2]
Fe_2O_3@C	Fe_2O_3 nanoparticles encapsulated within carbon shells and the appropriate voids are formed between Fe_2O_3 nanoparticles and carbon shells	Hydrothermal, sol-gel and calcination	Resorcinol–formaldehyde resin	548.9<br>413.2	250<br>300	0.2 A/g<br>1 A/g	[3]
Fe_2O_3@C_{PDA}	Fe_2O_3 nanorods with carbon-coated rod-type	Hydrothermal, sol-gel and calcination	Dopamine hydrochloride	603.2	1000	2 A/g	[4]

	nanostructures						
Fe_2O_3@NC	Fe_2O_3 nanoparticles surface encased in N-doped carbon	Precipitation, sol-gel and calcination	Polyvinyl pyrrolidone, 2-methylimidazole	782.9	300	1.0 A/g	[5]
Fe_2O_3@SCTPC	Fe_2O_3 nanosphere coated by carbon	Hydrothermal and calcination	Sulphonated coal tar pitches	1106.74<br>373.5	100<br>500	0.1 A/g<br>2.0 A/g	[6]
HPCs/Fe_2O_3-2	Fe_2O_3 nanoparticles embedded in hierarchical porous carbon matrix	Salt-template, freeze-drying, calcination and hydrothermal	Egg white	1357.6<br>472.3	100<br>1000	0.2 A/g<br>5.0 A/g	[7]
CNFs@C-Fe_2O_3	Fe_2O_3 nanocubes uniformly grown on carbon nanofibers	Electrospinning, crystallization, precipitation and calcination	Polyacrylonitrile	559.9	500	0.5 A/g	[8]
Fe_2O_3@CC	Fe_2O_3 nanowire array supported on carbon cloth	Hydrothermal and calcination	Carbon cloth	514.1	100	0.1 A/g	[9]
3D-CT@Fe_2O_3-NPs@C	3D lateral interconnected vertical and lateral carbon tube (CT) grid with Fe_2O_3 encapsulated in the wall of CTs	Chemical vapor deposition	Lateral carbon tube	896.2	700	1.0 A/g	[10]
Fe_2O_3@MPG	Fe_2O_3@microwave-puffed graphite sandwich structure	Precipitation, calcination and microwave treatment	Feasible spent graphite	632	500	2.0 A/g	[11]

Fe_2O_3@C	Fe_2O_3 nanoparticles dispersed in a carbon base	Hydrothermal, freeze-drying and calcination	Peanut shells	1000.8<br>573.5	100<br>200	0.2 A/g<br>1.0 A/g	[12]
BLC/Fe_2O_3-6	Fe_2O_3 nanoparticles anchoring on the carbon substrate	Heat-treatment	Longan pulps	626.6	1000	1.0 A/g	[13]
Fe_2O_3 NPs@PCSs	Fe_2O_3 nanoparticles embedded in a porous carbon sheets assembled hierarchical matrix	Salt-template, freeze-drying and calcination	Carboxy methyl cellulose	756.1<br>545.4	200<br>200	0.2 A/g<br>1.0 A/g	[14]
Fe_2O_3@C nanosheets	Conformal carbon coated ultrathin porous Fe_2O_3 nanosheets	Hydrothermal and calcination	Glucose	991.5<br>594.5	200<br>550	0.2 A/g<br>1.0 A/g	[15]

References

1. Mohebi, M., Bolghanabadi, N., Taghizadegan, P., Khoshnoudi, E. B., Khodabakhsh, M., & Simchi, A. (2025). In situ hybridization of α-Fe_2O_3 nanorods with a mesoporous carbon matrix for reversible lithium-ion batteries. Journal of Alloys and Compounds, 182200.
2. Yang, S., Zhao, N., Zheng, K., Sun, L., & Niu, J. (2025). Lithium storage behavior of expanded microcrystalline graphite/F Fe_2O_3 anode for lithium-ion batteries. ACS omega, 10(17), 17673-17683.
3. Hu, J., Wang, Z., Li, C., Lan, D., Yi, B., Tian, Q., & Yi, Z. (2025). A relatively simple preparation strategy of void-involved Fe_2O_3/C and its lithium storage. Journal of Energy Storage, 109, 115120.
4. Yang, H. W., Maniyazagan, M., Naveenkumar, P., Kang, N., Kang, W. S., & Kim, S. J. (2025). Superior electrochemical performance and structure Evolution of Fe_2O_3 nanorods via carbonized polydopamine for lithium-ion batteries. Journal of Electroanalytical Chemistry, 979, 118920.

5. Song, Y., Zhang, W., Zhang, H., Dong, Z., Li, Y., & Yao, Y. (2025). Facile and low-cost synthesis of core-shell Fe_2O_3@ NC as a high-performance anode material for Lithium-ion batteries. Available at SSRN 5333798.
6. Li, J., Miao, X., Han, B., Wang, K., An, B., Xu, G., ... & Zhou, W. (2024). Sulphonated coal tar pitch as a carbon source to develop the storage capacity of Fe_2O_3@ C materials. Catalysis Today, 439, 114822.
7. Liu, X., Xiong, K., Yuan, H., & Zhao, J. (2024). Effective combination of Fe_2O_3 with hierarchical porous carbon for high-performance lithium storage. Ionics, 30(3), 1373-1381.
8. Si, J., Zhao, M., Cui, Z., Cai, D., Zhan, H., & Wang, Q. (2024). Crystalline and amorphous Fe_2O_3 nanocubes grown on electrospun carbon nanofibers for lithium-ion batteries and lithium-sulfur batteries: A comparative study. Applied Surface Science, 657, 159782.
9. Yin, X., Liu, Z., Li, X., Qi, M., Hu, M., & Mu, X. (2024). Smart construction of Fe_2O_3 nanowire arrays on carbon cloth for enhanced supercapacitor and lithium-ion battery. Journal of Electrochemical Energy Conversion and Storage, 21(4), 041012.
10. Pan, Q., Zhang, S., Chen, B., Sun, S., Han, F., & Meng, G. (2024). Fe_2O_3 nanoparticles encapsulated in the inner walls of integrated 3D carbon tube grid as high-performance anode for lithium-ion batteries. Journal of Power Sources, 602, 234348.
11. Jia, P., Sun, J., Li, S., Wang, W., Song, Z., Zhao, X., & Mao, Y. (2024). A feasible recycling route for spent graphite: Microwave-assisted puffing with Fe_2O_3 loading to construct high-performance anode for lithium-ion batteries. Materials Today Sustainability, 25, 100620.
12. Wu., S., Jin., Y., Wang., D., Xu., Z., Li., L., Zou., X., Zhang., M., Wang., Z., Yang., H. (2023). Fe_2O_3/carbon derived from peanut shell hybrid as an advanced anode for high performance lithium ion batteries. Journal of Energy Storage, 68, 107731.
13. Liu., R., Liu., H., Yang., Q., Ma., Y., Dong., D., Wan., J. (2023). Longan-derived biomass carbon induced cubic-type ferric oxide nanoparticles for efficient lithium ion battery anode. Energy & Fuels, 37(21), 16979–16987.
14. Pan, Y., Luo, C., Yang, D., Sun, P., Chen, J., Sui, Z., & Tian, Q. (2023). Coordination chemistry-assisted ecofriendly preparation of Fe_2O_3 nanoparticles embedded in hierarchical carbon for improved lithium storage. Journal of Alloys and Compounds, 962, 171153.
15. Pan, Y., Luo, C., Yang, D., Sun, P., Chen, J., Sui, Z., & Tian, Q. (2023). Ultrathin porous Fe_2O_3@ C nanosheets: Novel preparation strategy and high lithium storage. Applied Surface Science, 635, 157763.

Table. S2. The fitted parameters based on the equivalent circuit

	**Before cycling**			**After 100cycles**		
	**5:5**	**7:3**	**8:2**	**5:5**	**7:3**	**8:2**
$\mathbf{R_s}$	4.3	4.6	5.3	3.2	5.8	6.6
$\mathbf{R_{ct}}$	36.6	45.8	161.1	180.5	22.1	8.5
$\mathbf{R_{SEI}}$	-	-	-	46.12	7.7	15.1
